# A comprehensive dataset of biomass and critical variables for *Verrucodesmus verrucosus* culture in bubble column photobioreactors

**DOI:** 10.1016/j.dib.2025.112003

**Published:** 2025-08-22

**Authors:** Hector Ricardo Hernandez-de Leon, Julio Cesar Martinez-Morgan, Jose Armando Fragoso-Mandujano, Hector Rodriguez-Rangel, Leonel Ernesto Amabilis-Sosa, Sheyla Karina Flores-Guirao

**Affiliations:** a Tecnológico Nacional de México/ I.T Tuxtla Gutiérrez/ Doctorado en Ciencias de la Ingeniería, Carretera Panamericana KM. 1080, Tuxtla Gutiérrez, 29050, Chiapas, Mexico; b División de Estudios de Posgrado e Investigación, Tecnológico Nacional de México/IT de Culiacán, *Av*. Juan de Dios Batiz No 310, Culiacán, 80220, Sinaloa, Mexico

**Keywords:** Sensor-based data collection, Experimental data acquisition, High-resolution measurements, Bioprocess data, Kinetic modeling, Environmental condition monitoring

## Abstract

The presented dataset accurately records biomass generation and readings of environmental variables that directly affect the growth of Verrucodesmus verrucosus crops in bubble column photobioreactors. The information was obtained through a monitoring system based on sensors and manual quantification of dry weight, which allowed the acquisition of physicochemical parameters such as irradiance, temperature (°C), nitrate concentration (NO₃), dissolved oxygen (DO), oxygen gas (O₂ gas), carbon dioxide gas (CO₂ gas), and electrical conductivity. This dataset is essential for creating and validating kinetic models and machine learning algorithms crucial for biomass estimation. It contains 1080 observations, serving as a vital resource for examining growth kinetics and creating highly accurate predictive models that aid in optimizing microalgae culture systems. Advanced preprocessing methods were employed to maintain data integrity and quality, such as outlier detection, data interpolation, and key feature extraction. The combination of different inference models, including multiple linear regression, second and third-degree polynomial models, and Gaussian regression, demonstrates the feasibility of predictive strategies for biomass quantification. In addition to its relevance for developing prediction models, this dataset facilitates reproducibility in studies on microalgae biomass. It drives the advancement of bioprocess engineering and environmental monitoring by generating high-quality experimental information.

Specifications Table

**Table:** 

Subject	Biology
Specific subject area	Microalgal biomass dataset, environmental monitoring, kinetic modelling, bioprocess data processing.
Type of data	Raw sensor data, processed data, analyzed data, tables.
Data collection	The dataset was obtained from experimentation with microalgae cultures in a bubble column photobioreactor. The samples were monitored using calibrated sensors to read the critical variables temperature, pH, irradiance, dissolved oxygen, nitrates (NO₃), oxygen gas (O₂), carbon dioxide gas (CO₂), and electrical conductivity. Biomass was quantified using the dry weight method. The data were preprocessed to detect outliers, using linear interpolation and extracting the necessary characteristics to generate the modeling of growth kinetics.
Data source location	Bioprocess Laboratory, Tecnológico Nacional de México, Campus Tuxtla Gutiérrez, Chiapas, México.
Data accessibility	Data are available with this article and also at Repository name: Mendeley data repository Data identification number: https://doi.org/10.17632/c7f34t83pd.2 Direct URL to data:https://doi.org/10.17632/c7f34t83pd.2
Related research article	None.

## 1 Value of the Data

• The dataset contains a structured record of biomass data for the species *Verrucodesmus verrucosus*, which allows researchers to analyze growth kinetics under controlled conditions and explore various applications in biotechnology.
• Monitoring crop growth using sensors and integrating manual biomass measurements using the dry weight method increases data reliability. It also facilitates the analysis of critical growth variables such as irradiance, pH, nitrate concentration, dissolved oxygen, oxygen gas, carbon dioxide gas, and electrical conductivity.
• Researchers can use the dataset to develop and validate predictive models, including kinetic growth models, statistical analyses, or machine learning models. These models are efficient for estimating biomass in microalgae cultures and allow for optimizing the production of microalgae in photobioreactors.
• This dataset provides valuable information related to the behavior of physiological mechanisms for microalgae utilization, such as nutrient uptake and carbon assimilation, metabolic adaptation strategies, and their implications for bioprocesses.

## 2 Background

Microalgae are indispensable elements in the production of biomass, generation of biofuels, and carbon absorption, representing a crucial role for industrial biotechnology [1,2]. The species Verrucodesmus verrucosus presents a high yield in biomass production in crops under controlled growing conditions (1.8 g/L) [3,4], superior to studied species such as Chlorella vulgaris (1.5 g/L) [5], and Spirulina platensis (1.3 g/L) [6]. Using bubble column photobioreactors for crop growth improves nutrient distribution, gas transfer, and physicochemical stability by establishing optimal growing conditions [7]. This system has exhibited a higher biomass production than other microalgae species under comparable growing conditions, highlighting its potential for large-scale production [8].

Biomass estimation continues to be challenging due to the various sampling techniques and measurement methodologies available [9]. The most commonly used gravimetric methods, such as dry weight quantification, provide reliable results but are limited due to their low temporal resolution. Monitoring using sensors facilitates the acquisition of high-resolution data, but it is necessary to maintain the calibration of the two positives to ensure measurement reliability [10,11]. The integration of these techniques allows the generation of structured data sets that could be used in predictive modeling applications [12].

This dataset integrates measurements obtained from monitoring carried out with cabled sensors and manual biomass measurements. It provides a suitable framework for modeling the kinetic growth of microalgae and its possible applications in machine-learning models [13]. The relationship between crop biomass generation and the operating conditions of the environmental growth parameters improves the reliability of the developed models and also contributes to the optimization of microalgae production systems [14].

## 3 Data Description

**Fig. 1:**
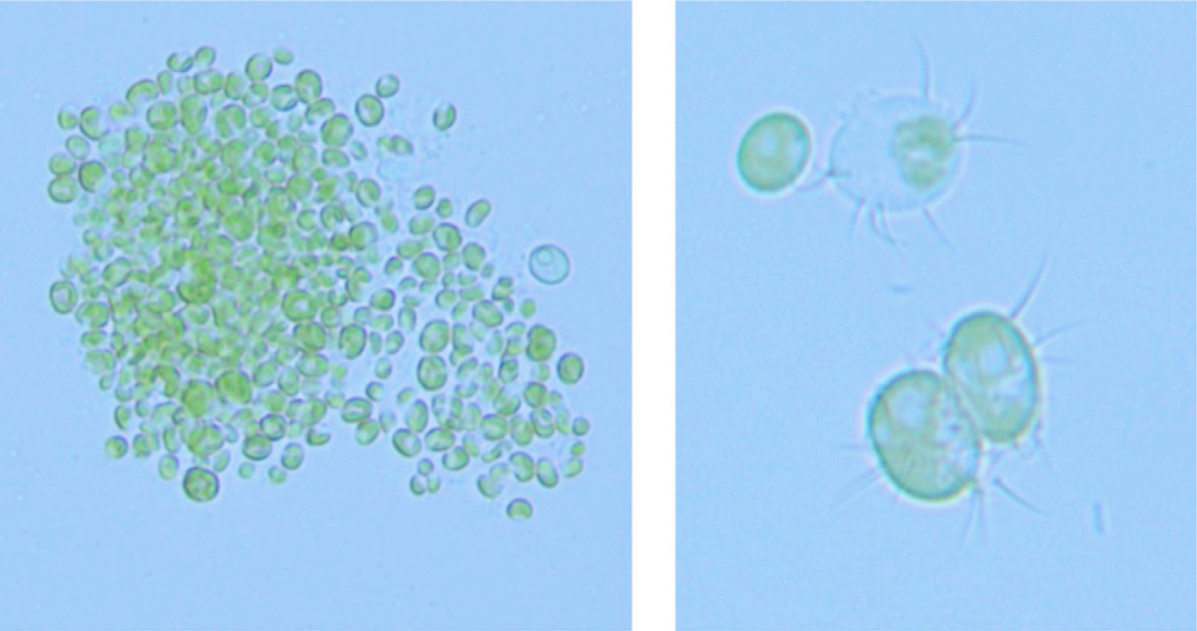
Microscopic images of *Verrucodesmus verrucosus* species.

The dataset provides the detailed quantification of biomass of Verrucodesmus verrucosus (Fig. 1) grown in a bubble column photobioreactor under controlled operation conditions for growth. The information presented contains direct biomass measurements and physicochemical parameter data readings obtained with calibrated sensors that allow kinetic analysis for growth and computational modeling. 1080 records are added that demonstrate the interactions between critical variables such as temperature (°C), irradiance, dissolved oxygen (DO), nitrate concentration (NO_3_), oxygen gas (O_2_ Gas), and carbon dioxide gas (CO_2_ Gas), and electrical conductivity, which are essential to perform biomass growth modeling.

Data acquisition was carried out by combining two complementary approaches; the variables were monitored using calibrated sensors, and biomass quantification was carried out using the dry weight method; this provided accurate data on the critical variables, facilitated the monitoring on the dynamics of the system, using these data as a reference to validate the biomass estimates. Preprocessing techniques were implemented to ensure data consistency, reliability and alignment, including synchronization of manually obtained and sensor-generated data, interpolation, feature extraction and outlier detection. These techniques enabled the identification and correcting of discrepancies derived from measurement frequencies and sampling methods, optimizing the data quality used predictive modeling.

The dataset presented specific interactions between critical variables and biomass production in crops. The concentration of nitrates reveals an inverse relationship with the accumulation of biomass, which indicates that during the growth phases of microalgae, the higher the consumption of nitrates, the higher the production of biomass is obtained. Keeping crop pH stable in the 7–9 range is associated with better biomass yields; if values are outside this range, there is a risk of damaging crops and inhibiting their growth. Irradiance and dissolved oxygen show the photosynthetic efficiency present in various species of microalgae. The observed patterns allow us to identify the most favorable conditions to optimize the cultivation processes at the experimental level.

## 4 Experimental Design, Materials and Methods

The experimentation was carried out with cultures of the microalgae Verrucodesmus verrucosus under controlled operating conditions in bubble column photobioreactors, to ensure accuracy and reproducibility in the biomass quantification process. This section presents the methodology carried out for the growth of the crop and the data acquisition procedure carried out to generate the dataset used for the estimation of biomass.

### 4.1 Inoculum preparation for the microalgae cultures

This study was conducted using the species Verrucodesmus verrucosus. The microalgae inoculum was prepared in the medium Blue Green 11 (BG-11) in 1 L graduated vials. The inoculum volume (225 mL) represents 10 % of the total operating volume of the photobioreactor, with an initial cell density of 1 × 10^6^ cells/mL. The samples were exposed to 2000 lx with a 12:12 light-dark photoperiod incubated for 12 days to promote growth.

### 4.2 Cultivation conditions

The microalgae were cultured in a bubble column photobioreactor with continuous aeration, with a volume of 2.25 L. The system was prepared to ensure uniform mixing and gas exchange, thus optimizing nutrient distribution and light penetration into the growing medium. The photobioreactor was illuminated with LED lights in a spectral range of 450 to 700 nm. The intensity of the light was maintained at 2000 lx with a photoperiod of 12:12 (light-dark). The microalgae were grown in BG-11 medium, a medium commonly used for the growth of these species. The pH was established in a range of 7–9, in order to avoid variations that would affect cell growth.

### 4.3 Data collection

The dry weight method was used to obtain the biomass measurements. In each measurement, 3 mL of culture was extracted for the analysis of the samples, the filtration was implemented on cellulose acetate membranes of 0.45 μm, dried at 75 °C for 24 h. Subsequently, the dry biomass was weighed using a precision analytical balance of ± 0.0001 g. Critical variables were monitored in culture samples with calibrated sensors, including irradiance, temperature, pH, nitrate concentration (NO₃), dissolved oxygen (DO), oxygen gas (O₂), carbon dioxide gas (CO₂), and conductivity (Table 1). Biomass measurements were carried out every 48 h per sample, with the sensors taking readings at 15-minute intervals, this variation in sampling frequency represents a challenge when integrating the data from the data, the measurements are not temporally aligned. To address this problem, temporal alignment was performed by robust interpolation (linear and polynomial), which linked the sensor data to the biomass sampling values. Potential discrepancies caused by the gradual loss of sensor accuracy over time (sensor drift) over the 30 days of crop growth and the lower variability resulting from manual methods for determining biomass were also mitigated through regular calibration of the sensors used and the application of standardized procedures for sampling and weighing. The measures taken ensured the accuracy and compatibility of both data sources.

**Table 1:** Structure summary.

Monitored variable	Unit
Biomass	g/L
Temperature	°C
Irradiance	µmol/m^2^/s
Nitrate concentration	mg/L
Oxygen gas	%
Carbon dioxide gas	ppm
Dissolved oxygen	mg/L
Conductivity	µScm^−1^
pH	0–14

### 4.4 Preprocessing

Data collection integrated a strategy that combines two different approaches. The first consisted of the continuous monitoring of culture samples using sensors, with records obtained in a growth kinetics lasting 30 days. The correct monitoring of the growth phases allowed the identification of environmental fluctuations observing significant variations in the critical variables. In a complementary way, another approach focused on the quantification of biomass through manual analytical sampling. The integration of both approaches included the detection of outliers using interquartile range thresholds, the interpolation of the data from the higher-frequency sensors to synchronize them with the values of the low-frequency biomass measurements, and the temporal alignment of both types of data was performed. Linear interpolation was chosen over spline methods due to its computational simplicity and the uniform behavior of the measurement data from the sensors, which allowed the validation of the data and facilitated the understanding of the behavior of the growth dynamics of microalgae for use in predictive modeling tasks.

### 4.5 Inference model

**Fig. 2:**
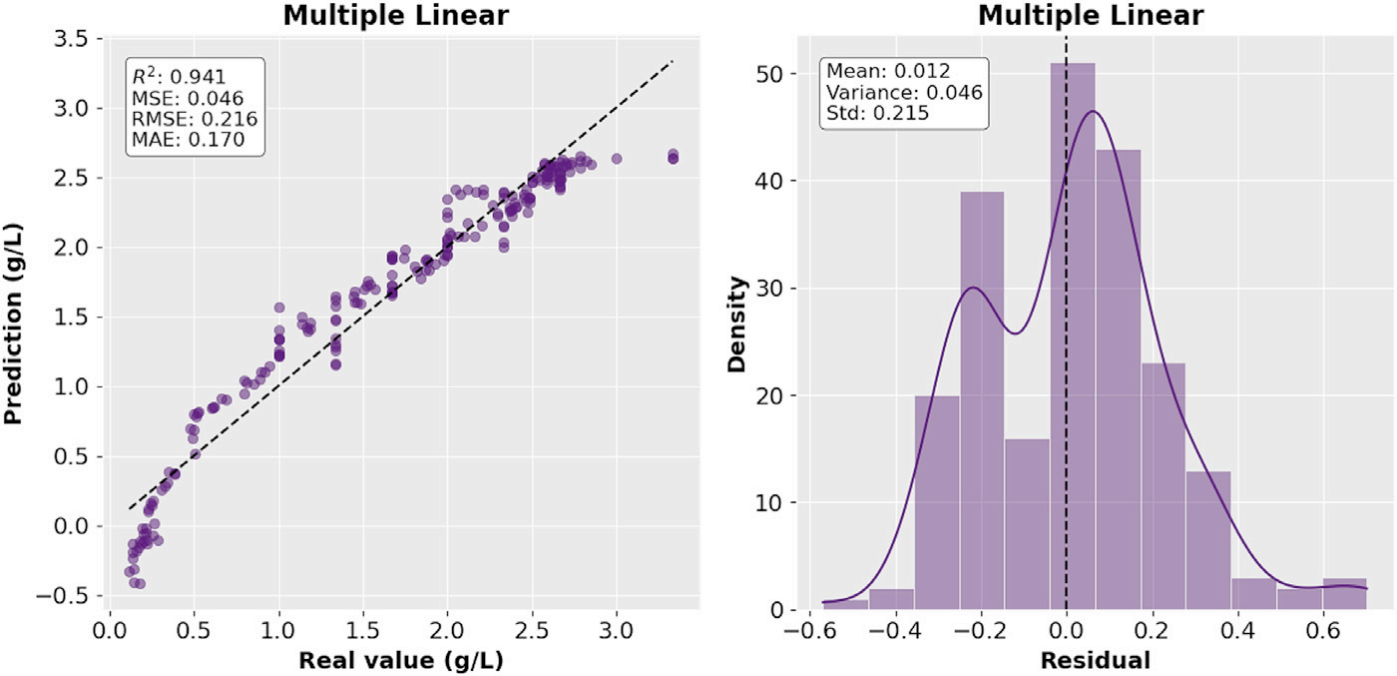
Multiple Linear Regression.

Four regression models were implemented for biomass estimation: Multiple Linear Regression (MLR), second- and third-degree Polynomial Regression, and Gaussian Process Regression (GPR). The models were selected to identify the correlation between the input variables and biomass production. The MLR model assumes a linear combination of predictors and uses it as a benchmark for comparison. Polynomial models add nonlinear elements; the third-degree polynomial captures the most complex trends to model nonlinearities within model patterns and is efficient when the relationships between variables are not clearly observed. Data scaling was applied to normalize the input variables and the data were divided in an 80 %−20 % ratio for training and testing respectively, ensuring the consistency of the information when comparing the results between each model. Fig. 2 demonstrates the performance of the MLR model by comparing predicted versus actual biomass values and the behavior of the residuals.

**Fig. 3:**
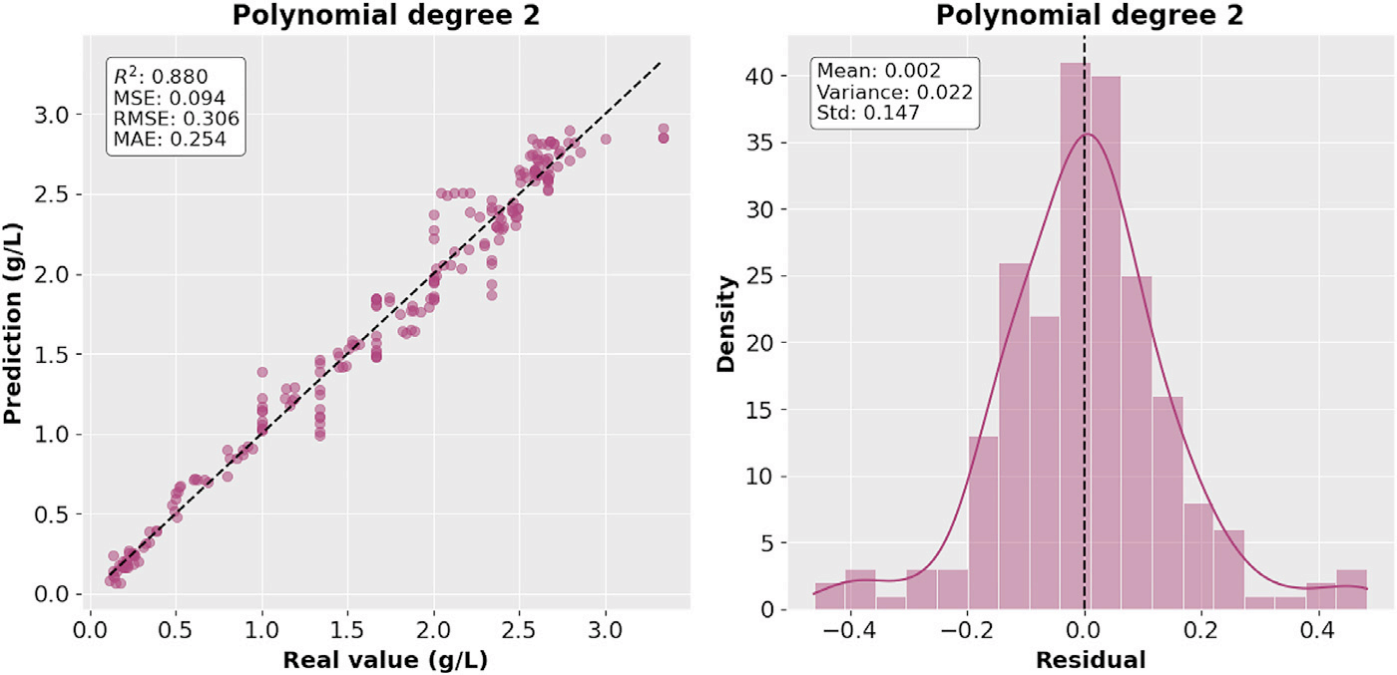
Polynomial regression degree 2.

**Fig. 4:**
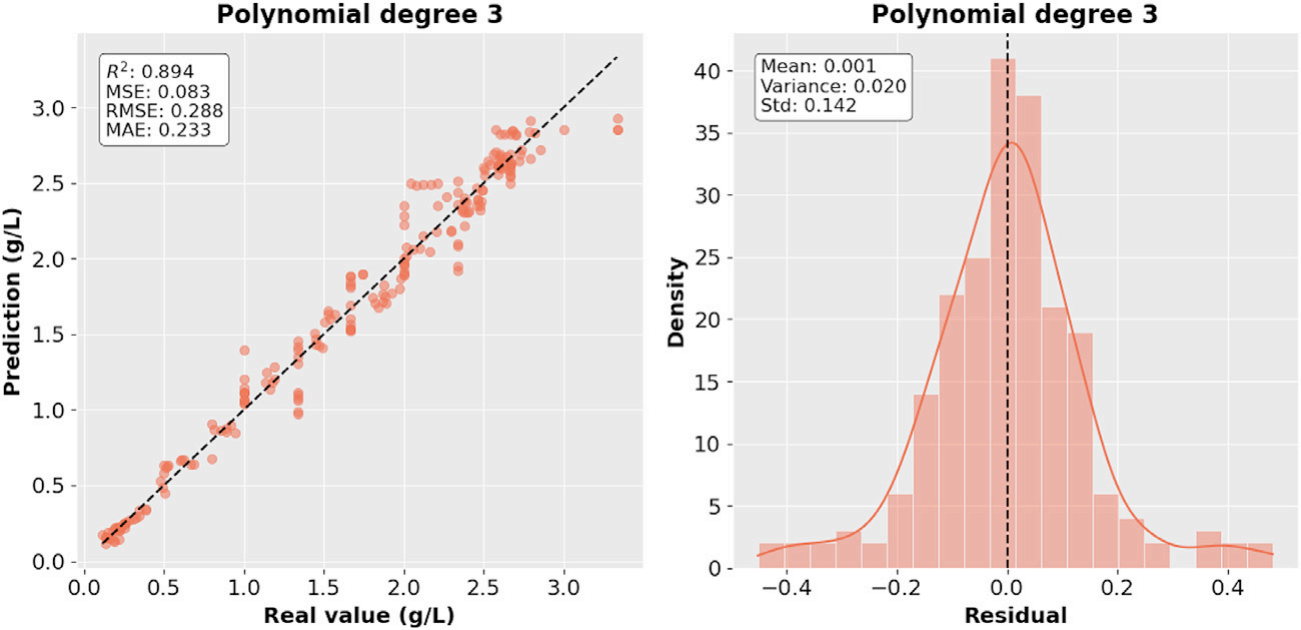
Polynomial regression degree 3.

Fig. 3 and Fig. 4 show the behavior of the data with polynomials of second and third degree respectively. The grade 2 model better represents the nonlinearity of the data; however, the grade 3 model reduces residual error by improving prediction precision.

**Fig. 5:**
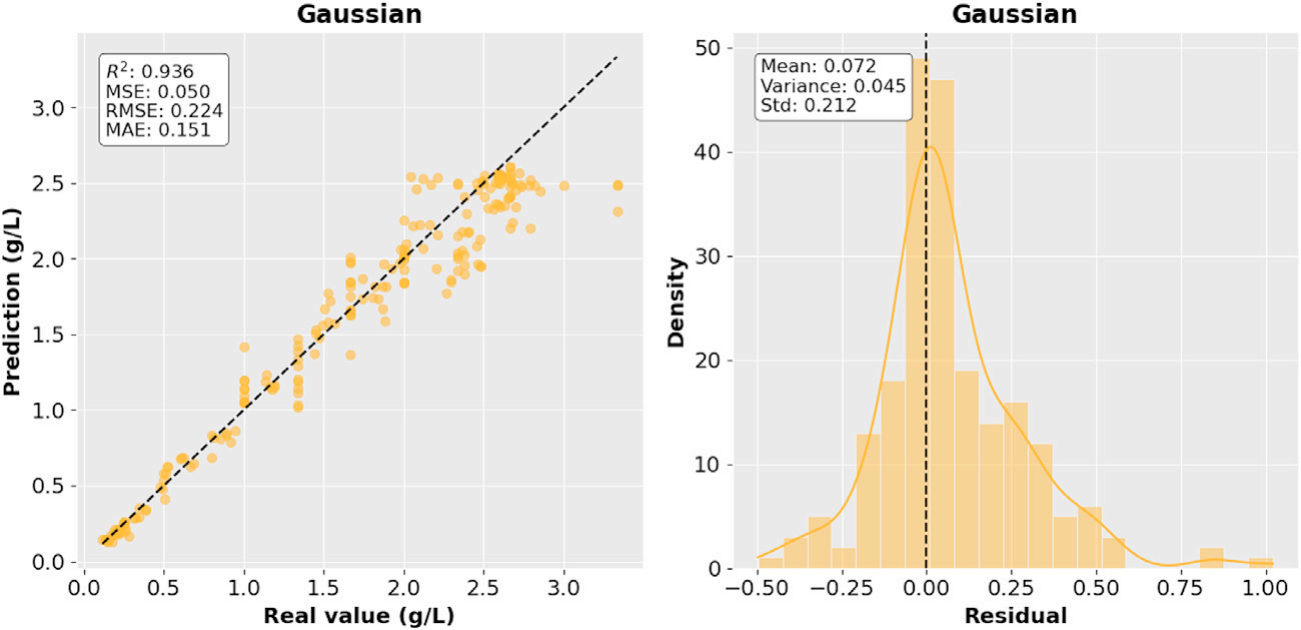
Gaussian Regression.

Fig. 5 shows the results of GPR model, in which its ability to identify significant variations in the nonlinear relationships of variables and model these complex patterns is observed.

### 4.6 Performance analysis

**Fig. 6:**
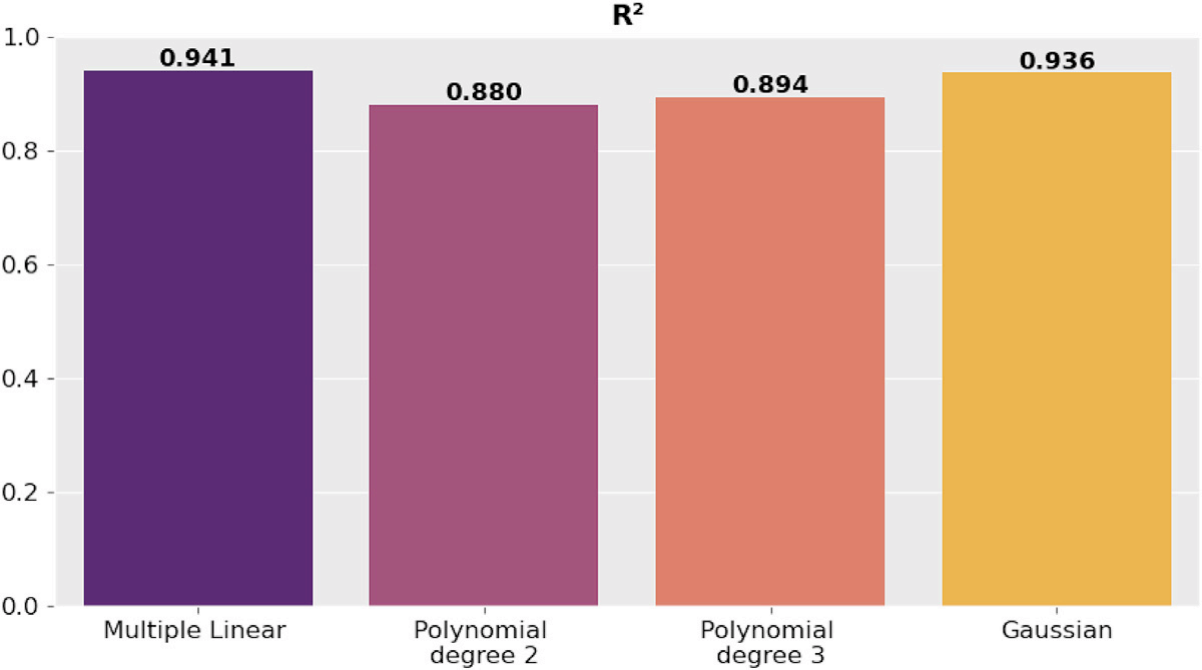
Comparison of R^2^ between the regression models.

**Fig. 7:**
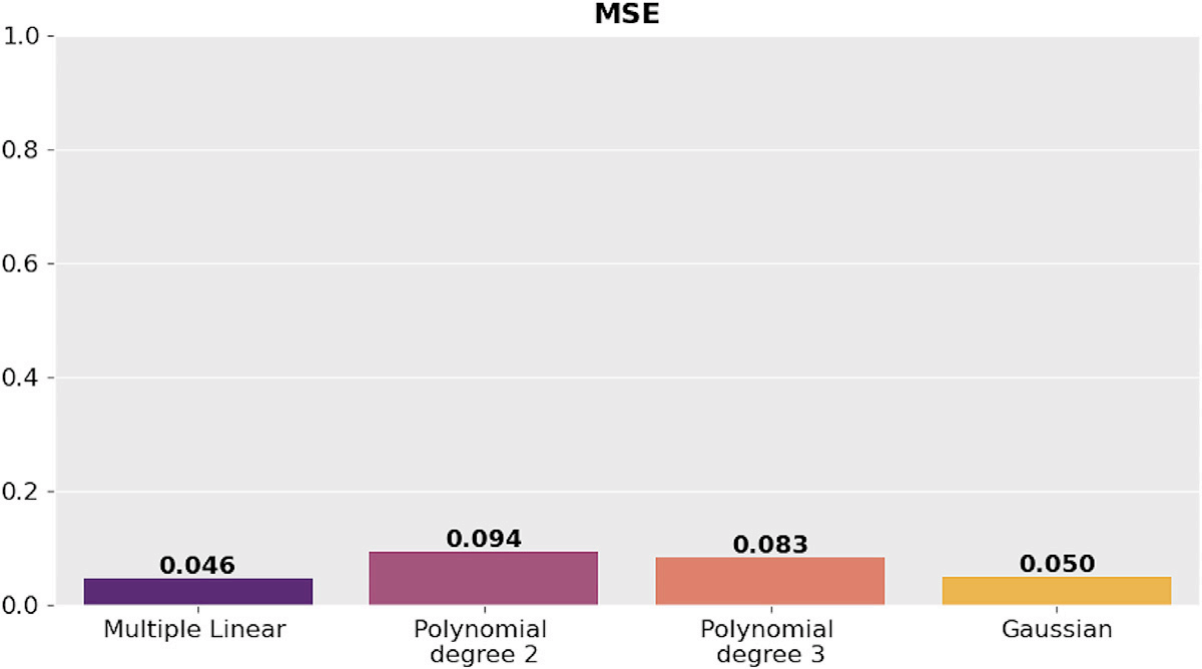
Comparison of MSE between the regression models.

**Fig. 8:**
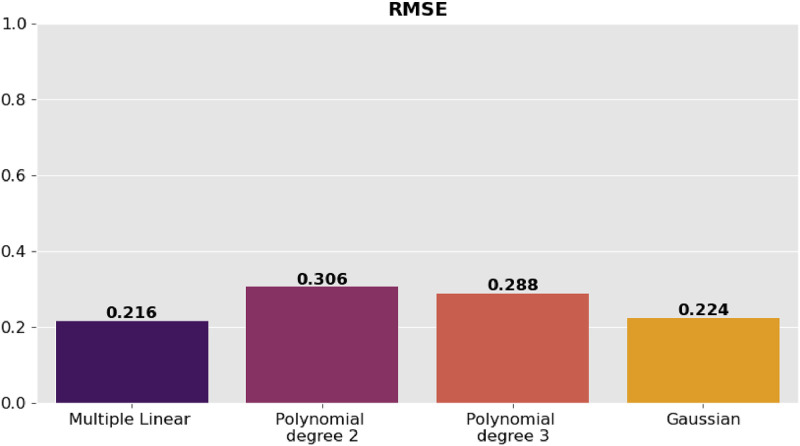
Comparison of RMSE between the regression models.

**Fig. 9:**
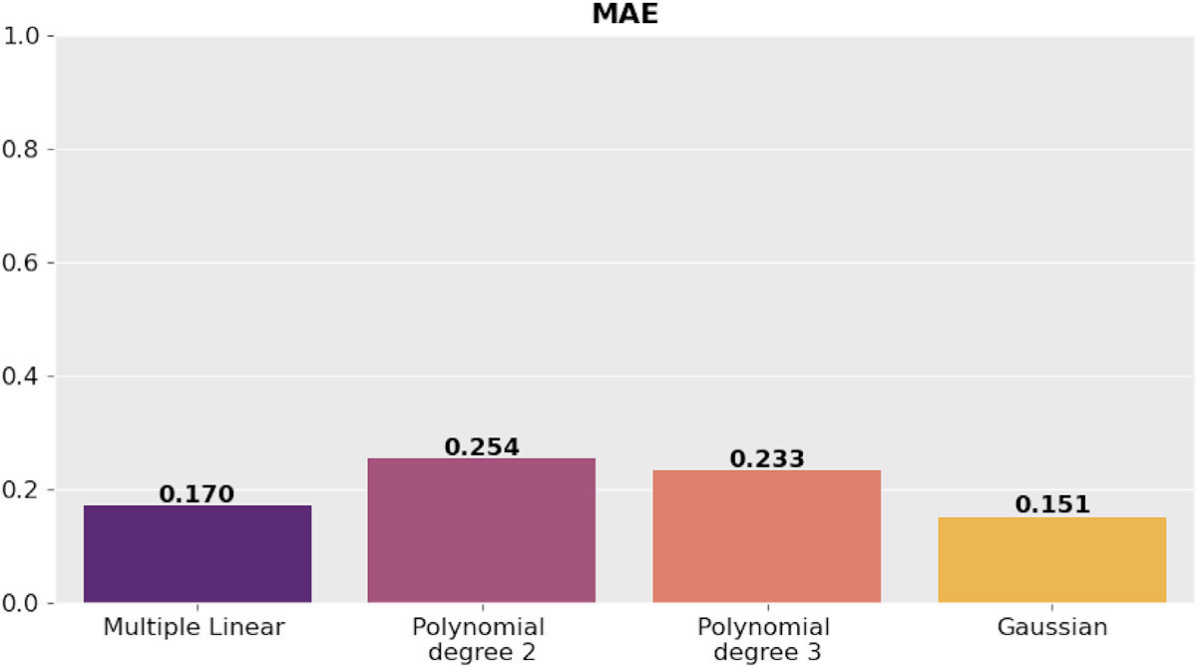
Comparison of MAE between the regression models.

The precision of the predictions was evaluated using the corresponding regression metrics such as mean square error (MSE), root mean square error (RMSE), mean absolute error (MAE) and coefficient of determination (R^2^). Table 2 presents the comparison of the results of the metrics for each model and they are shown graphically in Fig. 6, Fig. 7, Fig. 8–9.

**Table 2:** Model comparison.

Model	R^2^	MSE	RMSE	MAE
Multiple linear regression	0.941	0.046	0.216	0.170
Polynomial regression degree 2	0.880	0.094	0.306	0.254
Polynomial regression degree 3	0.894	0.083	0.288	0.233
Gaussian regression	0.936	0.050	0.224	0.151

**Fig. 10:**
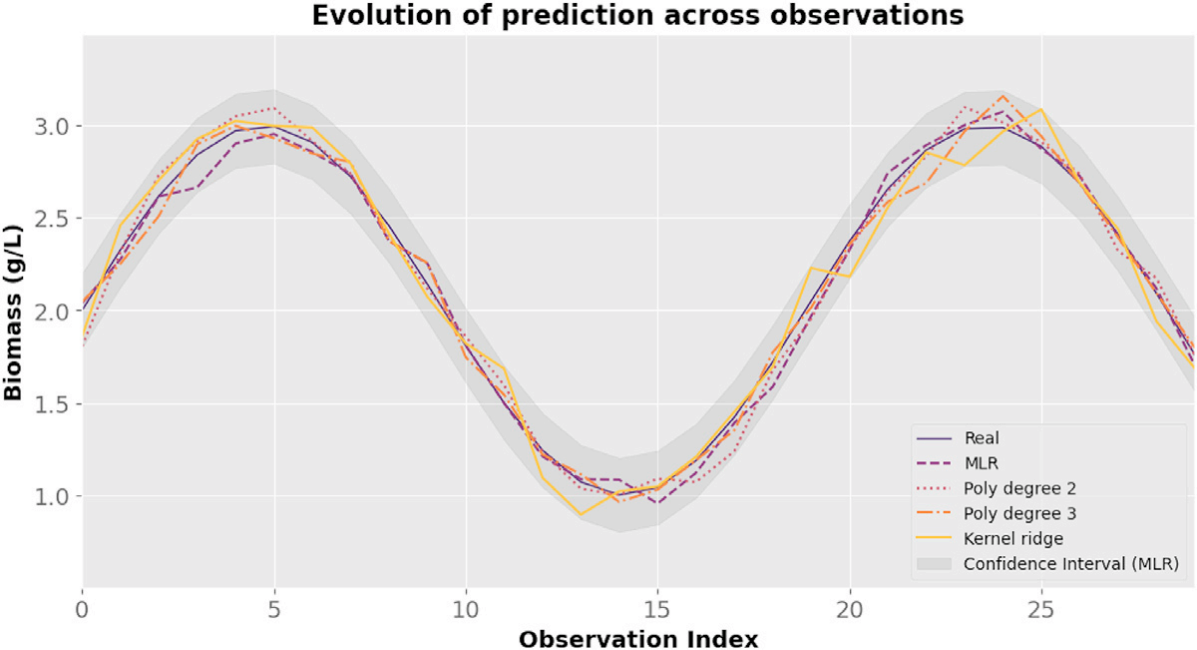
Evolution of biomass prediction across observations.

Fig. 10 demonstrates the behavior of biomass predictions through each observation, which facilitates the analysis of biomass growth dynamics over time.

In practical terms, MLR is easy to implement and interpret, making it suitable for conducting initial assessments when computationally resources are limited.

Polynomial regressions achieve moderate nonlinearities with precision, highlighting the third degree as the most precise compared to the second degree but with greater complexity.

GPR is the most flexible for modeling complex nonlinear relationships and manages to capture subtle patterns in data. However, it is more computationally expensive and requires adjustments in the parameters. These differences suggest that the selection of the regression model should consider a balance between the accuracy of the predictions, the available computational resources and, the variability in the operation commonly present in real scenarios with photobioreactors.

## Limitations

The dataset provides a detailed record of the growth of the microalgae Verrucodesmus verrucosus. However, there is a limitation caused by the discrepancy in the frequencies of manual measurements for the quantification of biomass and measurements with sensors. To integrate and align the two types of measurements, data interpolation techniques were required, which would consequently generate small uncertainties that affect the biomass estimate.

## Ethics Statement

The current work does not involve human subjects, animal experiments, or any data collected from social media platforms.

## CRediT authorship contribution statement

**Hector Ricardo Hernandez-de Leon:** Conceptualization, Investigation, Methodology, Resources. **Julio Cesar Martinez-Morgan:** Conceptualization, Methodology, Validation, Formal analysis, Writing – original draft, Supervision, Project administration. **Jose Armando Fragoso-Mandujano:** Writing – review & editing. **Hector Rodriguez-Rangel:** Methodology, Software, Investigation, Writing – review & editing, Visualization, Supervision. **Leonel Ernesto Amabilis-Sosa:** Methodology, Validation, Formal analysis, Data curation, Writing – review & editing, Visualization. **Sheyla Karina Flores-Guirao:** Methodology, Investigation.

## Data Availability

Mendeley DataA comprehensive dataset of biomass and critical variables for Verrucodesmus verrucosus culture in bubble column photobioreactors (Original data).
